# Correction: A control theory approach to optimal pandemic mitigation

**DOI:** 10.1371/journal.pone.0315749

**Published:** 2024-12-10

**Authors:** Prakhar Godara, Stephan Herminghaus, Knut M. Heidemann

In Fig 6, the red curves for *ρ/τ* = ∞ are incorrect. There should be no oscillations. Please see the correct Fig 6 here.

**Fig 6:**
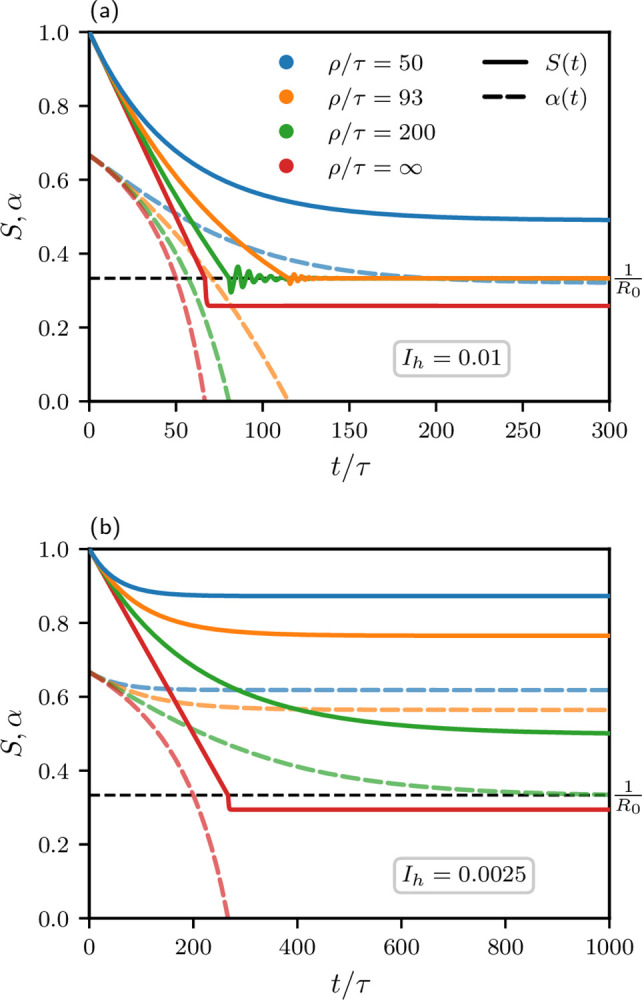
Typical pandemic scenarios for different average immunity loss times. **ρ/τ** ∈ **{50, 93, 200, ∞}, corresponding to curves from right to left (or see color code), and different values for I**_**h**_**, namely 0.01 in the top graph (a) and 0.0025 in the bottom graph (b).** Solid curves: *S*(t). Dashed curves: α(t). The fraction of acutely infected citizens is kept at I_h_ in phase II until herd immunity is reached (*S* = 1/R_0_, horizontal dashed line). If this is successful (if I_h_ > Î_h_, see Eq 30) phase III begins, i.e., mitigation measures are being released (α = 0). For finite immune response (ρ/τ < ∞), *S*(t) oscillates around its limiting value *S*_∞_ = 1/R_0_. In the limit of infinite immune response (ρ/τ = ∞), there are no oscillations and S(t) converges to S_∞_ = 1-R_∞_ = 1+W(exp(-1-R_0_I_h_)), with the Lambert W function (see also Fig 3). Other parameters: R_0_ = 3, τ = 10 days.
